# Supplementary material for: Ultrasound mediated delivery of quantum dots from a proof of concept capsule endoscope to the gastrointestinal wall
Source: Sci Rep. 2021 Jan 28;11:2584. doi: 10.1038/s41598-021-82240-1 (PMC7844260; doi:10.1038/s41598-021-82240-1)
Supplement: Supplementary file 1 — Supplementary Figures. [file 41598_2021_82240_MOESM1_ESM.pdf]

Supplementary Information

Ultrasound Mediated Delivery of Quantum Dots from a Proof of Concept Capsule Endoscope to the Gastrointestinal Wall

Fraser Stewart[1, 2*+], Gerard Cummins[*3+], Mihnea V. Turcanu[4], Benjamin F. Cox[5], Alan Prescott[1], Eddie Clutton[6], Ian P. Newton[1], Marc P.Y. Desmulliez[7], Maya Thanou[8] Helen Mulvana[9], Sandy Cochran[4] and Inke Näthke[1]

1 The University of Dundee, School of Life Sciences, Dundee, DD1 5EH, United Kingdom

2 The University of Strathclyde, Department of Electronic and Electrical Engineering, Glasgow, G1 1XQ, United Kingdom

3 The University of Birmingham, School of Engineering, Birmingham, B15 2TT, United Kingdom

4 The University of Glasgow, School of Engineering, Glasgow, G12 8QQ, United Kingdom

5 The University of Dundee, School of Medicine, Dundee, DD1 5EH, United Kingdom

6 The University of Edinburgh, The Roslin Institute, Edinburgh, EH25 9RG, United Kingdom

7 Heriot-Watt University, School of Engineering and Physical Sciences, Edinburgh, EH14 4AS, United Kingdom

8 Kings College London, Institute of Pharmaceutical Science, London, SE1 1DB, United Kingdom

9 The University of Strathclyde, Department of Biomedical Engineering, Glasgow, G1 1XQ, United Kingdom

*G.Cummins@bham.ac.uk

^ fraser.stewart.101@strath.ac.uk
+these authors contributed equally to this work

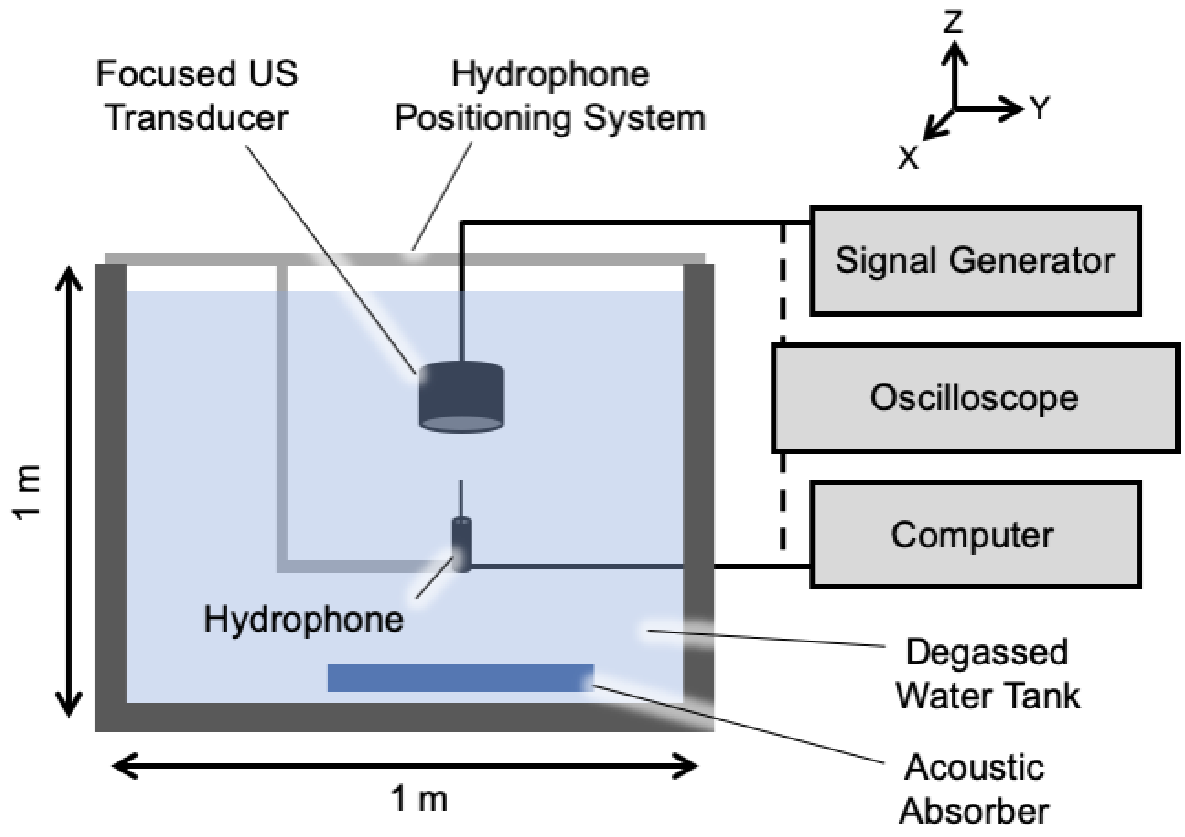

Supplementary Figure 1: Configuration of the ultrasound field mapping system with the needle hydrophone in the degassed water tank, centered and facing the active surface of the transducer.

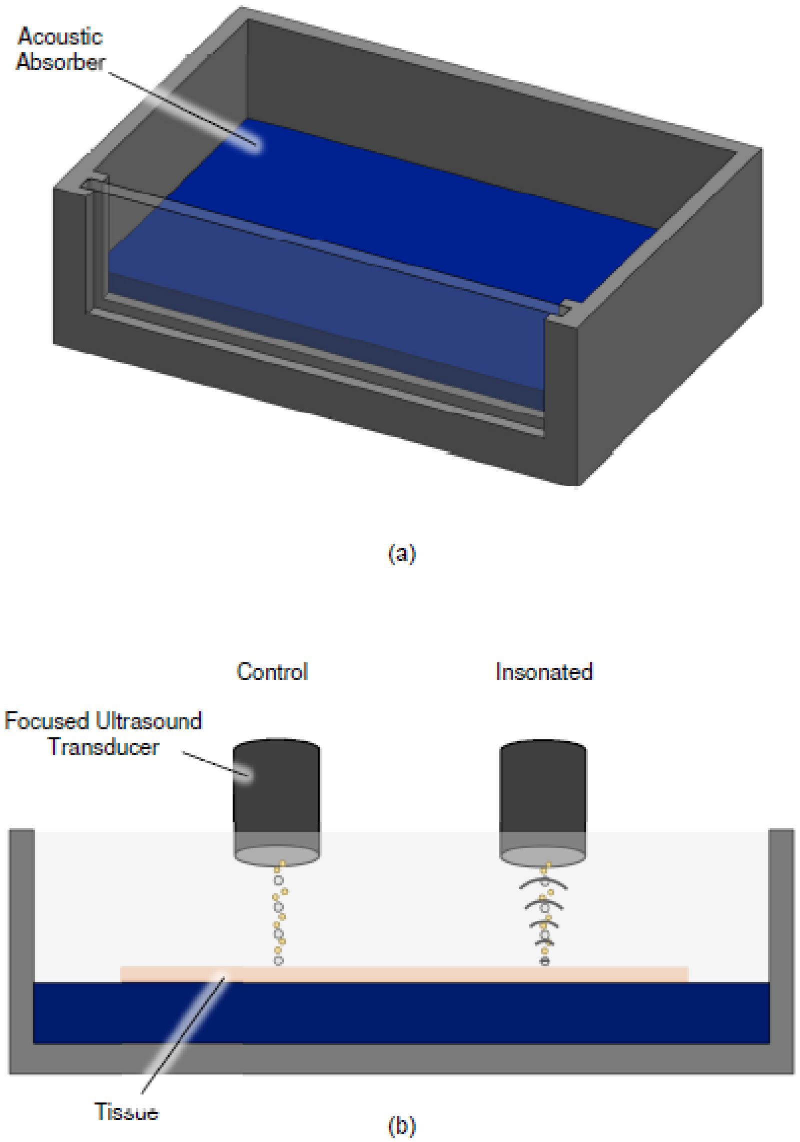

Supplementary Figure 2: (a) Insonation tank constructed with the same footprint as a standard multiwell plate. (b) Tissue was pinned to the acoustic absorber and the tank was filled with PBS. Experiments applied QDs without insonation (control, left), and QDs with insonation. Drawings not to scale.

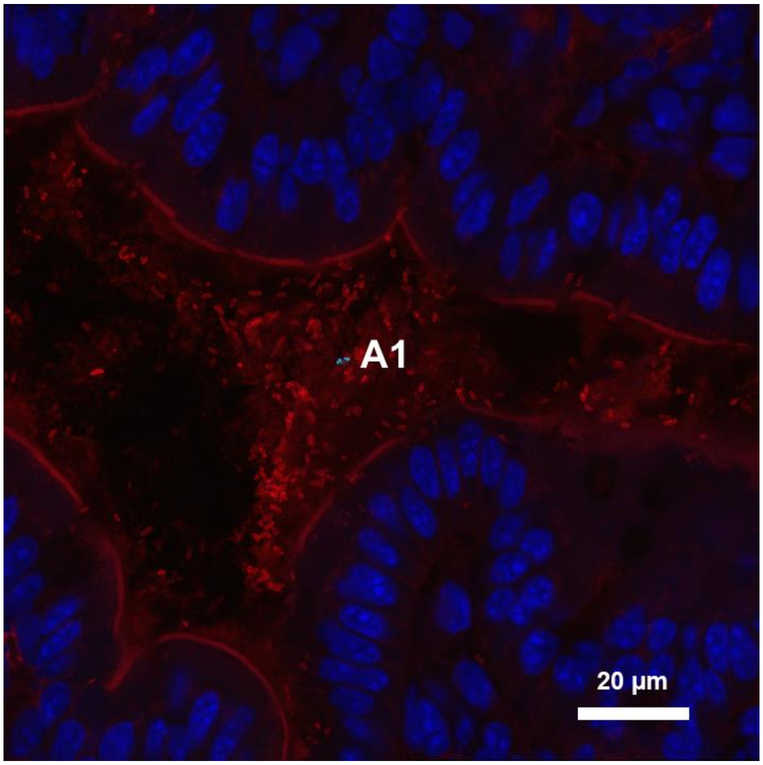

(a)

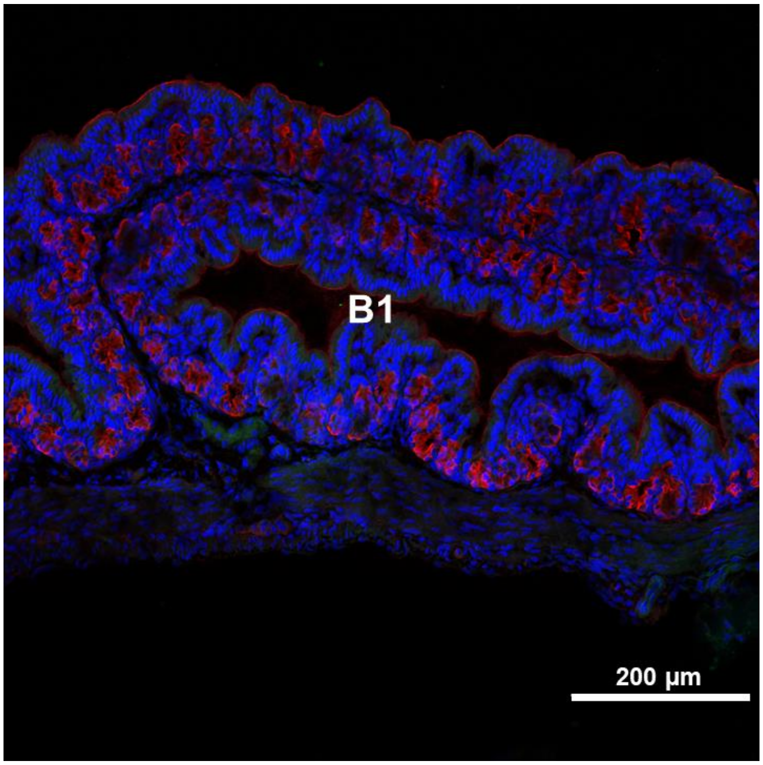

(b)

Supplementary Figure 3: Images showing staining of the murine colon and minimal presence of QDs. Images show errant QDs (green) are present on top of the mucosa layer (stained with WGA,

red) at A1 and B1 and did not penetrate the mucosa or the underlying intestinal tissue (marked by DNA stain to show nuclei, blue). Scale bars represent 20 μm in (a) and 200 μm in panels (b).
